# Facilitators and constrainers of civilian–military collaboration: the Swedish perspectives

**DOI:** 10.1007/s00068-018-1058-9

**Published:** 2018-12-12

**Authors:** Amir Khorram-Manesh

**Affiliations:** Department of Surgery, Institute of Clinical Sciences, Sahlgrenska Academy, Gothenburg University, Gothenburg, Sweden

**Keywords:** Civilian, Military, Healthcare, Collaboration, Facilitators, Constrainers

## Abstract

**Purpose:**

An increasing number of international and domestic armed conflicts, including terror attacks on civilians, along with constrained healthcare finance and resource limitation, has made a civilian–military collaboration (CMC) crucial. The purpose of this study was to identify facilitators and constrainers in CMC in a national perspective with a specific focus on medical aspects.

**Method:**

A literature review of recently published papers about civilian–military collaboration, along with a short survey, was conducted. For the review, major search engines were used.

**Results:**

The results indicated many facilitators, but few important constrainers with a high impact on the outcome. The conducted survey indicated discrepancies between the needs and resources.

**Conclusion:**

The current global and domestic security threats and challenges, make CMC critical and inevitable. However, there is a need for careful analysis of its consequences, impact, possibilities, and limitations to differentiate between our expectations and the current reality.

## Introduction

The increasing risk of natural and man-made disasters indicates a need for collaboration between different agencies in all aspects of emergency management [1–3]. Even though civilian–military collaboration (CMC) has a long history in various areas, their collaboration within the healthcare varies in different countries due to their international engagement, neutrality, and domestic needs [4–7]. Recent terror attacks on civilians worldwide, exhibits new unpredictable incidents, with variation in character, magnitude, target, and medical outcomes (severe combat-like injuries). Consequently, they also signal a need for raised preparedness, wider planning, and closer collaboration with the military healthcare [3, 7, 8].

Decades of financial constraint along with increasing sub-specialization in the medical profession has confronted the civilian healthcare with a shortage in emergency capacities (emergency and trauma staff and physicians) and truly challenges the outcome of any disaster or major incident [2, 9, 10]. Although the current situation may be comprehended as chaotic and disastrous, management of a large-scale incident requires both non-medical and medical capabilities. In general, any existing capability is a facilitator, while the lack of a capability is a constrainer. The existence or absence of valid disaster/emergency plan, trained and experienced command and control staff, reliable and unified communication system, ability to collaborate and coordinate, information-sharing, internal and external logistic, and training in a multidisciplinary setting are all important non-medical facilitators or constrainers. Meanwhile, evidence-based medical guidelines, simple and unified triage system, evidence-based prehospital, hospital, and on-route treatment strategies, are all important medical factors for successful medical management of an incident and consequently facilitators or constrainers [2, 9, 11–24].

From Napoleon’s warfare to today, the modern medical field’s assessment and management of injured patients have developed steadily. During the Korean War, MASH (Mobile Army Surgical Hospital) units, offered dialysis to patients with acute renal failure and repaired injured vessels. The results were a significant reduction in the mortality rate compared with that of World War II and a validation of the new strategy [25, 26]. In Vietnam, important advances in combat casualty care such as the rapid helicopter transport of casualties, the creation of dedicated trauma centers, minimization of post-trauma renal failure through crystalloid resuscitation, and the use of mechanical ventilators to support patients with acute respiratory distress syndrome were made. These advances and facilitators have been translated into civilian practice and constitute historical evidence of fruitful CMC in peace- and wartime [4–8, 27]. Over the centuries, the military has used staff from civilian healthcare to provide care to the wounded soldiers. In return, civilian healthcare staff have contributed with their gained knowledge to the development of trauma and emergency care [8, 25].

A collaboration between civilian and military healthcare organizations is beneficial for both parties and results in an exchange of experience, skills, resources, information, and improves the communication and integration of both systems. There is, however, a need for maintaining the development and competencies even in non-combat periods and in countries with no independent military healthcare, such as Sweden.

Recent global security challenges including terror attacks on civilians have signaled a change of the game by bringing the battlefield to the civilian backyard, indicating a need for an increased civilian preparedness and a new partnership with the military at home [2, 9, 10, 28, 29]. The incidents in Boston and Paris resulted in recommendations regarding some requirements needed for a successful incident management [2, 28]. Unified or compatible prehospital and hospital triage, existing mass casualty and blood delivery plans, short ambulance transport time, prehospital use of tourniquets and clotting devices, mutual communication system, psychological follow-up, and annual training, were all named as important factors necessary for a successful outcome after an incident. However, the main question appeared to be whether any European healthcare organization alone could fulfill these requirements? As for Sweden, any incident with the same magnitude as in Paris will be a devastating and yet stronger indication for CMC. In an earlier report, the non-medical aspects of Swedish CMC were discussed [11], the aim of this review was to identify facilitators and constrainers of a civilian–military collaboration in a global and national perspective with a focus on its medical consequences.

## Method

This study was conducted in two steps.

1. Survey: a questionnaire was designed based on the recommendations published after Boston and Paris terror attacks [2, 3]. It was distributed among 18 experts from 14 European countries (including Sweden) with many years’ experience within the field of emergency and disaster management and armed conflicts. It aimed to find out the current situation in their countries regarding; (1) unified prehospital triage, (2) compatible prehospital and hospital triage, (3) short ambulance transport time, (4) existing mass-casualty plans, (5) plans for blood delivery, (6) prehospital use of tourniquets, (7) prehospital use of clotting devices, (8) mutual communication system, (9) psychological follow-up, and, (10) annual training.
2. Review: a review of existing publications within the last 25 years (1993–2018) was conducted. Three search engines; PubMed, Scholar and Scopus were used with the focus on CMC and its consequences. Following keywords were used alone or in combination; civilian–military collaboration, medical aspects, non-medical aspects, medical outcome, mass casualty, terror attacks, types of injury, type of explosives, surge capacity, resource- and information-sharing.

## Results

### The survey

Fourteen out of 18 experts replied to our survey (78%). Contact was taken repeatedly with none respondents with no success. The questions, participant countries and results are presented in Table 1. The result showed that none of the countries included in this survey were fully ready for the next disaster. There was a lack of unified triage method. The use of tourniquets and clotting devices was controversial. There was neither a common communication system nor psychological follow-up and an annual training program was missing.

**Table 1:** Questions checking mass call readiness, sent to 18 experts from 14 countries, response rate 14/18 or 78%

Q	1	2	3	4	5	6	7	8	9	10	11	12	13	14	15	16	17	18
1	Yes	Yes	No	No	No	–	No	Yes	–	Yes	Yes	Yes	–	No	No	No	Yes	–
2	Yes	No	No	Yes	No	–	No	No	–	Yes	No	Yes	–	No	Yes	Yes	No	–
3	12 min	25 min	10–15 min	70 min	10–15 min	–	10 min	6 min	–	10 min	15 min	?	–	?	10–20 min	?	8 min	–
4	Yes	Yes	Yes	Yes	Yes	–	Yes	Yes	–	Yes	Yes	Yes	–	Yes	Yes	Yes	Yes	–
5	Yes	?	Yes	No	Yes	–	Yes	No	–	No	Yes	?	–	No	Yes	?	Yes	–
6	N/Y	Yes	Yes	Yes	No	–	Yes	Yes	–	Yes	Yes	Yes	–	No	No	?	Yes	–
7	No	No	No	No	No	–	No	No	–	Partly	No	?	–	No	No	No	?	–
8	Yes	Yes	No	No	No	–	No	No	–	No	Yes	Yes	–	?	Yes	Yes	Yes	–
9	Yes	Yes	No	Yes	Yes	–	Yes	No	–	No	Yes	No	–	No	Yes	Yes	Yes	–
10	No	Yes	No	Yes	No	–	Yes	No	–	Yes	Yes	No	–	No	No	No	Yes	–

Questions: (1) Do you have a mutual triage method on scene (prehospital) in your country? (2) Do you have the same triage method at hospitals? (3) What is the average ambulance transport time, within the city, from the scene of an event to the hospital? (4) Do your prehospital and hospital entities have a mass-casualty plan? (5) Do your blood banks have a mass-casualty plan? (6) Do your prehospital staff use tourniquets? (7) Do your prehospital staff use clotting devices? (8) Do you have a common communication system prehospital and hospital, which enables communication between all involved organizations? (9) Do you have a plan for psychological follow-up of your staff after a disaster/major incident/terror? (10) Do you have training/exercise for your staff annually? How many times?

Participants experts: (1) Belgium (MD, Trauma), (2) Croatia (MD, Trauma), (3) Denmark (MD, Trauma), (4) France (MD, Trauma), (5) Germany one (MD, Disaster), (6) Germany two (MD, Anesthesiology), (7) Italy one (MD, Anesthesiology), (8) Italy two (MD, Anesthesiologist), (9) Italy three (MD, General Surgeon), (10) Netherland (MD, Trauma), (11) Norway (MD, Trauma), (12) Portugal (MD, Trauma), (13) Romania (MD, Trauma), (14) Slovenia (MD, Trauma), (15) Spain (MD, Trauma), (16) Sweden one (MD, Trauma), (17) Sweden two (MD, Anesthesiology), (18) United Kingdom (RN, Emergency)

### Review

Of total 61,900 publications, duplicates, those related to collaboration between military and other organization, e.g., voluntary organizations, those related to humanitarian interventions, peace-keeping operations, and other fields of civilian–military collaboration, were excluded. Of the remaining 150 publications, those not relevant to the main topic or only dealing with non-medical aspects of CMC (*n* = 96), were also excluded. The remaining 56 publications were finally used as references in this article. The contents and information in the articles were then categorized into following subgroups: prehospital and hospital care (triage, specific medical measures), and treatment.

### Prehospital and hospital care

An emergency plan based on risk and vulnerability analysis is a mandatory measure for a prepared organization. It consists of necessary non-medical steps to initiate a leadership, collaboration, coordination, mutual communication, staff recruitment, and annual training. A good plan may also facilitate some medically important measures such as blood delivery, unified triage, psychological follow-up and quick distribution of patients to the operating theatres, intensive care units, or other wards [2, 9, 11]. A reliable, quick, standardized and professional prehospital healthcare, including aeromedical and marine care, is an important part of the disaster, and emergency care [30–32]. The extent of aeromedical [30] and marine [31] healthcare depends on the geography of a land and its landscape. Both aeromedical and marine healthcare require globally unified advanced knowledge. Although there are different prehospital care philosophies (Franco-German vs Anglo-American, etc.), the basic knowledge among the staff and the model of practice are also globally unified and similar in both civilian and military organizations within the same nation [32]. Nevertheless, there might be some differences in routines and procedures that may threaten patients’ safety and the outcome of a mission such as different nomenclature, abbreviations, and reporting routines [11]. These discrepancies can be overcome using standardized protocols and education. MIMMS (Major Incidents Medical Management and Support) is a standardized course, used in many countries [33]. The strength of the concept is its military nature and international spreading, which enables global collaboration.

### Triage

In an emergency with many injuries, triage will be decisive for the balance between needs and resources. The prehospital and hospital triage methods must be simple, unified or compatible [2]. Triage must be conducted by the most senior doctors since the outcome may have a high impact on resources and total management of the incident [2, 9, 11–13]. There are many triage methods for prehospital and hospital use, most of which are adopted, modified or adjusted based on institutional needs and conditions [34–36]. In an isolated and local incident, the type of triage method may not be an important factor; however, in a national or international perspective, diverse triage methods and discrepancy in their outcomes may result in an increased morbidity and mortality [35, 37]. Prehospital triage aims at quickness and simplicity (using a simple method to quickly transfer the patient), while hospital triage aims at clinical justice and efficiency (to ensure that the patient receives the appropriate level and quality of care, using resources effectively) [2, 38, 39]. Such complexity in obtaining a safe and correct diagnosis using technological devices seems to be contradictive in a mass casualty situation, where a simple and quick method is preferred to save lives [2, 40]. Triage should also exhibit a continuity, i.e., to be compatible in all levels of decision-making to alleviate the flow of the patients into the system in a local, regional or national perspective. In this perspective, there is a lack of compatibility between prehospital and hospital entities, which impacts the outcome negatively.

### Specific medical measures

#### The concept of anesthesiologic damage control

Anesthesiologic damage control, using anesthesia and fluid resuscitation, is integral to the damage control approach in trauma patients with ongoing hemorrhage and can have a profound impact on the patient’s outcome. The process aims to optimize hemostasis and long-term survival and rapid transport of the patient to diagnostic studies and surgery. It also includes measures necessary to avoid recurrent shock, and may further benefit the patient by the careful, logical administration of analgesics and sedatives. The concept is important for the care of unstable trauma patients and was proven to be highly useful in the prehospital management of victims in Paris attack [2, 41].

#### The use of tourniquet

This has been and still is a controversial measure at the prehospital level. However, evidence obtained from the literature review indicates that its use should not be overlooked in the presence of potentially life-threatening hemorrhage despite the potential risks involved in its utilization. It is recommended that emergency medical staff, both civilian and military, should be trained in and equipped for the proper use of tourniquets in appropriate situations such as a mass casualty incident for temporary control of hemorrhage while the situation is brought under control [42].

#### The use of clotting material/devices

The use of hemostatic agents is one of the latest measures for the emergency control of traumatic hemorrhages and is reported to decrease the associated mortality and morbidity. However, the search for the perfect hemostat is an ongoing process and its prehospital use is not fully developed in the civilian setting [43].

### Treatment

Although different etiology, the extremity injuries are common in both civilian and military setting. While most of the civilian injuries are caused by blunt trauma, over 80% of extremity injuries in a combat are caused by blasts and high-velocity gunshot wounds [44–46].

Blast injuries are caused by rapid pressure waves created by the detonation of explosives and cause multisystem, life-threatening injuries in single or multiple victims simultaneously. Indoor explosions cause the most severe injuries and have the worst outcomes. Survivors have predominantly primary and tertiary blast injuries. Secondary blast injuries may mainly occur in suicide bombings in open and/or semi-confined spaces. Life-threatening injuries involve lungs and hollow viscera. Limb injuries are rare in civilian setting and are mostly caused by a secondary blast effect created by projectiles and shrapnel implanted in explosive devices. Blast injury associated with skeletal damages may involve multiple skeletal sites and organ systems. Non-operative management and damage control techniques together with tertiary surveys to identify missed injuries are part of the treatment regimen [2, 3, 47–49].

The “Low”- or “High”-velocity energy delivered by a gunshot, results in a multimodal injury sustained to the vital organs. The impact of the bullet (damages) on tissues is characterized by a cavitation process or direct delivery of energy. Muscles, bone, and blood vessels are mainly affected in the limbs. Almost all high-energy gunshots are considered contaminated and should be treated accordingly. Stabilization of bone, soft tissue care, adequate wound coverage, and restoration of limb function are important parts of the treatment strategy. Bone loss and soft tissue coverage together with maintenance of limb alignment and joint congruency restoration in cases of severe comminution are the big challenges [3, 46–49].

Although civilian injuries cannot be compared in complexity to those sustained in a military combat, their absolute numbers are higher. Consequently, clinical studies and the development of technologies and treatment policies are best conducted in a civilian setting. Furthermore, the discrepancies in the number of patients may have an impact on the skills of the surgeon. While military surgeons manage most complex injuries, they may have less training in times of low military casualty flow. Civilian surgeons, on the other hand, have a higher rate of civilian injuries, but less knowledge in treating complex combat injuries [45].

## Discussion

This report aimed to identify facilitators and constrainers for a CMC (civilian–military collaboration). The result emphasizes a need for collaboration between the two healthcare organizations, but it also indicates a need for careful analysis of medical and non-medical aspects of such collaboration to differentiate the expectation from the reality.

After a period of global demilitarization and financial constraint, many countries faced with the demands on cutting military and ever-increasing healthcare budgets. For countries such as Sweden, it resulted in saving on what can seldom happen, i.e., wars and disasters. Nevertheless, the new era of violence, terror, and unrest, has obliged both the civilian and military organizations to develop capacities for flexibility, and collaboration and a new approach to unity, resource- and information-sharing [2, 28, 50].

The current terrorist threats have made collaborative medical training between military and civilian agencies crucial [6]. The complexity of injuries and the discrepancies in experience between civilian and military healthcare systems is another important indication for CMC. New efforts to integrate National Trauma Care System by integrating Military and Civilian Trauma Systems have already been initiated to achieve zero preventable deaths after Injury. Research and clinical practice between both partners are needed to establish best practices for treating severely injured military and civilian population, including other important areas such as triage, the concepts of anesthesiologic and surgical damage control, etc. [2, 45, 51]. Equally important is the exchange of lessons learned in both organizations as a foundation for developing new technologies and improved treatment strategies to be used in subsequent conflicts [45].

To enhance the common capacities of both organizations, in addition to the skills and competency, there is a need for a mutual meeting, exercises and training [52, 53]. Currently, there are professional trauma and emergency courses that are available for physicians, nurses, and medics. Advanced Trauma Life Support (ATLS) for physicians, Advanced Trauma Care for Nurses (ATCN), ATLS-Operational Emphasis (ATLS-OE), Prehospital Trauma Life Support Course (PHTLS), Tactical Combat Casualty Care (TCCC), Advanced Trauma Operative Management (ATOM), Advanced Surgical Skills for Exposure in Trauma (ASSET), and IATSIC courses in Damage Control Surgery and Anesthesia (DSTC and DSATC) are some existing courses. Other similar courses may also exist in other countries. However, some of these courses are resource-consuming and expensive and demand a multicenter collaboration [8], and others need to have a current content with input from both military and civilian experts to ensure relevancy to both environments [52, 53].

Civilian and military organizations have different mandates during an armed conflict. While civilian healthcare endeavor to provide life-saving assistance to affected populations, friends or enemy, the military’s task is to maintain security and uses all its power to protect own before others [5]. This may create ethical and security issues when the resources are scarce, regarding the care of enemy vs. own, the responsibility for the safety and security of patients, population, soldiers and medical facilities, and the use or storage of the weapon. Thus, forging these two organizations should be planned and tested long before any major incident and without compromising civilian control of security policy or undermining military effectiveness [29]. Despite little experience, civilian physicians might be expected to participate, if not lead, medical operations and make vital decisions and the task can be too difficult without previous experience and mental preparedness [54]. Consequently, it is important to offer educational initiatives that make all involved professionals aware of each other’s capabilities and limitations and encompasses other important issues such as legal, political, ethical, cultural, and traditional perspectives in disaster and emergency management. Recent developments in simulation courses offer new opportunities to include these aspects and involve different agencies into the same scenario, towards the same goal in a changeable and safe environment, where mistakes can be made, information and resources can be shared, and collaboration can be achieved. MRMI (Medical Response to Major Incidents) is one of these courses [55]. Similar courses may exist elsewhere.

In a Swedish perspective, preparedness and simplicity is the key to success. Creating a flexible surge is to increase the capacity in a simple, but effective way. The Swedish healthcare infrastructure, staff competency, and willingness to help are all winning factors [3, 11, 12]. A common healthcare system facilitates one routine and one structure in a friendly environment, where civilian’s high working load and excellent medical skills, together with the military staffs’ gained knowledge from international engagements, will offer a fruitful CMC in both hospital and prehospital environments. The exchange of knowledge between both partners enables research, education, and further technological development.

One major constrainer is the ever-increasing healthcare privatization with no attachment to disaster management, which also actualizes an important question about private clinics ‘social and national responsibility during emergencies’. Besides hierarchical differences in each organization (formal military and informal civilian leadership), another important constrainer for a Swedish CMC is the discrepancy between the civilian and military organization models at the prehospital level [11, 12]. The civilian healthcare abandoned the concepts of MIMMS to create a nationally adjusted prehospital concept. The new organizational model is based on important time-dependent and measurable quality indicators for command, control, and decision-making [56]. However, with the Swedish military still using MIMMS, the possibility of international assistance, and the lack of time during a disaster, the compatibility of two strategies remains to be verified and confirmed. One way to compensate for this shortcoming is to offer a MRMI–MIMMS course as a prelude to the full MRMI course. MIMMS explains in more detail the role of gold, silver, and bronze and MRMI compliments this by allowing to put the key roles into practice.

Another constrainer is the lack of unified and compatible triage method. The existing prehospital triage seems to be united across the country, but it differs from the national disaster triage, and the one used in some, if not many emergency departments. Switching the triage system from one daily method to the one used in disasters and major emergencies seem to be risky. Misunderstanding and misuse of one or another can cause severe damage, especially if one color or one word stands for different priorities in each triage method [37]. There is an ongoing debate about which triage method should be used at the Swedish emergency departments. The outcome will impact the prehospital and hospital compatibility discussion.

Medical guidelines, treatment policies, and educational initiative do not differ from what is currently practiced globally and are important facilitators. However, the lack of experience and opportunity to use them might be a serious constrainer. Many years in peace does not facilitate a good medical familiarity with new types of injuries. Common educational initiative, simulation exercises, and war surgery courses are needed. Additionally, an armed conflict brings up new security and ethical issues, which should be highlighted, discussed, and mentally trained, or otherwise they create a chaos which would have a negative impact on the medical outcome of an incident [3, 11, 16].

Although none of the participants’ countries in our survey fulfilled the requirements presented by expertise [2, 28], one recent idea might be to focus on individuals already on site as occasionally there are a few minutes between the time an incident occurs until the first responders arrive at the scene. Video reports from real incidents show many people on the scene either trying to do something for injured victims or are standing by helplessly. Therefore, this question has evolved on how and what these “Immediate Responders” can do to help the victims in a right way while waiting for qualified help. A list of different measures from CPR (Cardio Pulmonary Resuscitation) to triage can be discussed. Although this question cannot be answered at this moment, it actualizes the need for public education.

## Limitations

There are some limitations to this study:

1. The study is mainly based on English and in some cases Swedish, and German publications. Some important information published in other languages may have been missed.
2. The author used only three search engines and may also have missed other publications available by other search engines.
3. The survey was small and limited and only indicates a trend. A larger investigation is recommended.

## Conclusion

In conclusion, the multi-agency approach and management of disasters and emergencies are inevitable. The current global and domestic security threats and challenges demonstrate a new trend of increasing unpredictable terror attacks, characterized by severe combat-like injuries. Consequently, a collaboration between civilian and military healthcare is critical and necessary. Some factors such as similar organizations, traditions, unified goal, and similar situational picture facilitate such collaboration, while diverse routines (triage, reporting system, etc.), and the lack of experience may be serious constrainers. The sum of these factors demonstrates the gap and difference between expectations and reality. To fill this gap, there is a need for collaboration, mutual meetings, discussions, research, and educational initiatives, which not only aim to the individual competency but the competency and knowledge of all involved agencies. For Sweden and other similar countries, it is necessary to establish devoted research and educational centers, which not only offer specific professional courses but also multi-agency-inspired courses to spread the spirit of sharing experience and knowledge and to be prepared for the unpredicted in peace and war.
